# *In vitro *growth inhibition of bloodstream forms of *Trypanosoma brucei *and *Trypanosoma congolense *by iron chelators

**DOI:** 10.1186/1475-9292-5-3

**Published:** 2006-08-16

**Authors:** Karin Merschjohann, Dietmar Steverding

**Affiliations:** 1 Department of Parasitology, Ruprecht-Karls-University, 69120 Heidelberg, Germany; 2 Biomedical Research Centre, School of Medicine, Health Policy and Practice, University of East Anglia, Norwich NR4 7TJ, UK

## Abstract

African trypanosomes exert significant morbidity and mortality in man and livestock. Only a few drugs are available for the treatment of trypanosome infections and therefore, the development of new anti-trypanosomal agents is required. Previously it has been shown that bloodstream-form trypanosomes are sensitive to the iron chelator deferoxamine. In this study the effect of 13 iron chelators on the growth of *Trypanosoma brucei*, *T. congolense *and human HL-60 cells was tested *in vitro*. With the exception of 2 compounds, all chelators exhibited anti-trypanosomal activities, with 50% inhibitory concentration (IC_50_) values ranging between 2.1 – 220 μM. However, the iron chelators also displayed cytotoxicity towards human HL-60 cells and therefore, only less favourable selectivity indices compared to commercially available drugs. Interfering with iron metabolism may be a new strategy in the treatment of trypanosome infections. More specifically, lipophilic iron-chelating agents may serve as lead compounds for novel anti-trypanosomal drug development.

## Background

*Trypanosoma brucei *and *T. congolense *are the causative agents of sleeping sickness in humans and nagana in cattle, respectively. The protozoan parasites live extracellularly in blood and tissue fluids of mammals and are transmitted by the bite of infected tsetse flies (*Glossina *spp.). Over 60 million people living in 36 sub-Saharan countries are threatened with sleeping sickness [1] and 48000 deaths were reported in 2002 [2]. In addition, 46 million cattle are exposed to the risk of contracting nagana and the disease costs an estimated 1340 million USD per year [3]. Chemotherapy of African trypanosomiasis still relies on drugs developed decades ago and some of these display serious toxic side effects [4,5]. In addition, drug resistance in African trypanosomes is increasing [6,7]. Thus, new strategies to treat African trypanosomes are required.

In contrast to mammalian cells, bloodstream-form trypanosomes require only small amounts of iron for growth [8]. The reason for this is that bloodstream-form trypanosomes lack cytochromes and contain only four iron-dependent enzymes: aconitase, alternative oxidase, ribonucleotide reductase and superoxide dismutase. Recently, it has been shown that incubation of *T. brucei *bloodstream forms with the iron chelator deferoxamine results in growth inhibition of the parasite [9]. The compound does not inhibit iron-containing enzymes directly but acts by chelating cellular iron thus preventing its incorporation into newly synthesised apoproteins [9].

Here we investigated the trypanocidal activity of 13 chelators known to be able to complex iron ions against bloodstream forms of *T. brucei *and *T. congolense in vitro*. For comparison, the general cytotoxicity of the compounds was assayed with human myeloid leukaemia HL-60 cells.

## Results

The anti-trypanosomal activities and the general cytotoxicities of chelators were evaluated using the Alamar Blue^® ^assay [10,11]. For each reagent, the 50% inhibitory concentration (IC_50_) value, i.e. the concentration of a compound necessary to reduce the growth rate of the cells by 50% of that of controls, was determined. With the exception of 5-sulfosalicylic acid and dimethylglyoxime, all other compounds displayed anti-trypanosomal activities, with IC_50 _values varying 100-fold (Table 1). Generally, *T. congolense *was somewhat less susceptible to the compounds than *T. brucei*. A similar observation was recently made for the anti-trypanosomal activities of alkaloids [11]. The most trypanocidal chelators were deferoxamine (Desferal^®^), 1,10-phenanthroline and its 4,7-diphenyl and 2,9-dimethy-4,7diphenyl (bathocuproine, a Cu^1+ ^chelator) derivatives, and 8-hydroxyquinoline with IC_50 _values in the micromolar range. Thus, the anti-trypanosomal activities of these compounds are approaching those of commercial drugs used to treat sleeping sickness (suramin: IC_50 _= 0.4 μM) and nagana (diminazene aceturate (Berenil^®^): IC_50 _= 0.5 μM) previously determined for bloodstream forms of *T. brucei *427-221a and *T. congolense *STIB910 under identical experimental conditions [11].

**Table 1:** IC_50 _values of iron chelators for bloodstream forms of *T. brucei *427-221a and *T. congolense *STIB910, and for human myeloid leukaemia HL-60 cells, and IC_50 _ratios of cytotoxic to trypanocidal activities of the chelators.

Compound	IC_50 _(μM)			IC_50 _ratio	
		
	*T. brucei*	*T. congolense*	HL-60 cells	*T. brucei*/HL-60	*T. congolense*/HL-60
Deferoxamine	3.3	3.4	97.0	29.4	28.5
2,3-Dihydroxybenzoic acid	220	n.d.^†^	n.d	n.d.	n.d
Ethylenediamine-di-*o*-hydroxyphenylacetic acid	120	n.d.	325	2.7	n.d.
5-Sulfosalicylic acid	>1000 *	>1000 *	>1000 *	1	1
Tropolone	12.5	18.7	6.2	0.5	0.3
5-Methyl-tropolone	15.7	20.0	31.1	2.0	1.6
2,2'-Bipyridine	46.2	67.0	28.3	0.6	0.4
2,4,6-Tris(2-pyridyl)-1,3,5-triazine	28.6	75.0	90.1	3.2	1.2
1,10-Phenanthroline	3.3	5.3	8.5	2.6	1.6
4,7-Diphenyl-1,10-phenanthroline	2.0	4.5	48.3	24.2	10.7
Bathocuproine^‡^	3.0	>10 *	>10 *	>3.3	1
8-Hydroxyquinoline	2.1	206	7.7	3.7	0.03
Dimethylglyoxime	>100 *	>100 *	>100 *	1	1
Quercetin	16.3	62.6	>100 *	>6.1	>1.6

*The highest concentration tested.

^†^n.d., not determined.

^‡^Cu^1+ ^chelator.

Except for 5-sulfosalicylic acid, bathocuproine, dimethylglyoxime and quercetin, all other chelators were also active against HL-60 cells, with IC_50 _values ranging from 6.2 μM to 97 μM (Table 1). However, the IC_50 _ratios of cytotoxic/trypanocidal activity (selectivity index) were found to be in a modest range for all compounds (Table 1). Only deferoxamine (Desferal^®^) and 4,7-diphenyl-1,10-phenanthroline gave IC_50 _ratios between 10 and 30 (Table 1). For comparison, commercially available drugs used for treatment of sleeping sickness and nagana have significant higher selectivity indices (suramin: IC_50 _ratio = 1944; diminazene aceturate: IC_50 _= 692 [11]).

## Discussion

Compounds with a high affinity for iron are common in nature, especially in micro-organisms. Numerous iron chelators, so-called siderophores, have been isolated from bacteria and fungi [12]. Deferoxamine is such an iron chelator produced by the bacterium *Streptomyces pilosus*. It has been developed into the drug Desferal^® ^which is used for the treatment of acute iron poisoning and chronic iron-overload. In addition, deferoxamine has been shown to exhibit trypanocidal activity against bloodstream forms of *T. brucei *[9]. Here we demonstrated that deferoxamine is not only active to *T. brucei *but also to *T. congolense *bloodstream forms.

The isolation of siderophores in sufficient quantities for clinical applications is difficult and expensive. Therefore, other iron chelators which can be synthetically produced have been investigated in this study. 2,3-Dihydroxybenzoic acid and ethylenediamine-di-*o*-hydroxyphenylacetic acid are such synthetic iron chelators which have been used in patients with β-thalassaemia major [13] and to induce iron-deficiency in micro-organisms [14], respectively. However, both compounds only exhibited a weak trypanocidal effect. 5-Sulfosalicylic acid is another compound which complexes iron ions over a wide pH range but showed no toxic effect for trypanosomes and mammalian cells. The low anti-trypanosomal activities of these compounds may be due to their water solubility. Similarly, a reduced trypanocidal activity was recently observed for water-soluble derivatives of the DNA topoisomerase inhibitor camptothecin [15].

In contrast to water-soluble chelators, hydrophobic compounds displayed much better anti-trypanosomal activities. Tropolone is a lipid solubilizing agent for cationic metals and has been shown to release iron from cells [16]. It exhibited moderate trypanocidal activities but was also very toxic for mammalian cells. Its 5-methyl derivative showed similar anti-trypanosomal actions but with reduced cytotoxicity. 8-Hydroxyquinoline is a well-known lipophilic iron chelator [17] and is used as a disinfectant. It was very effective in killing bloodstream forms of *T. brucei*, however, no prediction can be made regarding its mechanism of action because of the lack of selectivity for metal ions. Quercetin is a flavonoid with metal chelating properties. It was only moderately toxic to trypanosomes. This may be due to its antioxidant properties [18] counteracting its trypanocidal effect as a chelator depriving the cell of iron. 2,2'-Bipyridine is a membrane-permeant, intracellular Fe^2+ ^chelator and has been shown to inhibit DNA virus replication by blocking the incorporation of iron into viral apoprotein ribonucleotide reductase [19]. However, 2,2'-bipyridine displayed only some trypanocidal activity like the related compound 2,4,6-tris(2-pyridyl)-1,3,5-triazine. On the other hand, 1,10-phenanthroline, a 2,2'-bipyridine derivative with increased lipophilicity and reduced free rotatability, substantially inhibited the growth rate of both trypanosomes and mammalian cells. The 4,7-diphenyl derivative of 1,10-phenanthroline showed a similar trypanocidal activity as its parent compound but was less cytotoxic. However, the anti-trypanosomal action of phenanthrolines seems not to be by depleting the parasites of iron. Against this is the observation that Fe^2+ ^and Cu^1+ ^complexes of 4,7-diphenyl-1,10-phenanthroline display similar trypanocidal activities (data not shown). In addition, bathocuproine (2,9-dimethyl-4,7-diphenyl-1,10-phenanthroline), another lipophilic phenanthroline derivative that selectively complexes Cu^1+ ^with no affinity for Fe^2+^, was also similarly toxic to trypanosomes. It seems that the lipophilicity of the compounds is the crucial factor. It is possible that phenanthrolines incorporate into membranes or intercalate into DNA and induce structural changes. In support of this, it has been shown that Fe^2+ ^complexes involving 1,10-phenanthroline and 4,7-diphenyl-1,10-phenanthroline bind to DNA [20].

## Conclusion

Iron deprivation may be a new strategy for the treatment of African trypanosomiasis. Although the compounds investigated in this study are not suitable for clinical use, our results suggest that lipophilic iron-chelating agents have a potential as novel anti-trypanosomal drugs. This finding may also be exploited in the future by utilizing the wealth of information currently being generated in the development of cell-permeable iron chelators as cancer chemotherapeutic agents [21-23]. In addition, future efforts should also aim at improving the selectivity of iron chelators. Moreover, iron chelators may be of interest for combination therapy with existing anti-trypanosome drugs.

## Materials and methods

### Reagents

Alamar Blue^® ^was from BioSource (Camarillo, CA, USA); EMEM with L-glutamine and without phenol red and foetal bovine serum were from Life Technologies (Eggenstein, Germany); RPMI 1640 without L-glutamine and without phenol red was from Bioproducts (Heidelberg, Germany); goat serum was from Boehringer Mannheim (Mannheim, Germany). Desferal was obtained from Ciba-Geigy (Wehr, Germany). All other chelators were from Sigma (Deisenhofen, Germany) or Fluka (Buchs, Switzerland). The Fe^2+ ^complexes of 1,10-phenanthroline and 4,7-diphenyl-1,10-phenanthroline, and 5-methyl-tropolone were kindly provided by Professor Michael Wink (Institute of Pharmacy and Molecular Biotechnology, Ruprecht-Karls-University, Heidelberg, Germany).

### Cells

Bloodstream forms of *T. brucei *TC221 was derived from the stock 427 [24] and was obtained from Prof. Peter Overath (Max-Planck Institute für Biologie, Tübingen, Germany). The animal origin of the *T. brucei *stock 427 cannot be definitely clarified anymore. Bloodstream forms of *T. congolense *STIB910 was cloned from *T. congolense *STIB249 which was isolated from a lion in Tansania in 1971 [25]. *T. congolense *STIB910 was kindly provided by Prof. Reto Brun (Swiss Tropical Institute, Switzerland). Human myeloid leukaemia HL-60 cells were obtained from the Deutsche Sammlung von Mikroorganismen und Zellkulturen GmbH (Braunschweig, Germany).

### Cell culture

Bloodstream forms of *T. brucei *and *T. congolense *were grown in Baltz medium [26] (EMEM plus 25 mM HEPES, 2.5 mM glucose, 1 mM Na-pyruvate, 0.05 mM hypoxanthine, 0.001 mM thymidine, 0.04 mM adenosine, 0.025 mM bathocuproindisulfonic acid, pH 7.5, 10 ml/l nonessential amino acids (100×)) supplemented with 16.7% heat-inactivated foetal bovine serum and goat serum, respectively, and 0.2 mM 2-mercaptoethanol. Human myeloid leukaemia HL-60 cells were propagated in RPMI 1640 medium supplemented with 2 mM L-glutamine and 10% heat-inactivated foetal bovine serum. All cultures were maintained in a humidified atmosphere containing 5% CO_2 _at 37°C (*T. brucei *and HL-60 cells) or at 34°C (*T. congolense*).

### Toxicity assays

Cells were seeded into 96-well tissue culture plates in 200 μl medium containing various concentrations of chelators dissolved in H_2_O (deferoxamine, 2,3-dihydroxybenzoic acid, ethylenediamine-di-*o*-hydroxyphenylacetic acid, 5-sulfosalicylic acid, tropolone, 5-methyl-tropolone, 2,2'-bipyridine, 1,10-phenanthroline), 99% ethanol (2,4,6-tris(2pyridyl)-1,3,5-triazine, 4,7-diphenyl-1,10-phenanthroline, 8-hydroxyquinoline, dimethylglyoxime, quercetin), or 100% DMSO (bathocuproine). The controls contained the respective solvent alone. In all experiments, the final solvent concentration was 1% which had no effect on cell growth. To ensure that the cells were in logarithmic growth phase during the entire experiment, they were seeded at an initial density of 1 × 10^4 ^*T. brucei*/ml, 4 × 10^5 ^*T. congolense*/ml, and 1 × 10^5 ^HL-60/ml, respectively. After 24 h (trypanosomes) or 43 h (HL-60) incubation, 20 μl of the colorimetric viability indicator Alamar Blue^® ^was added to each well. The cells were incubated for a further 24 h (trypanosomes) or 5 h (HL-60) so that the total incubation time was 48 h. Then, the plates were read on a Dynatech MR5000 ELISA reader (Denkendorf, Germany), using a test wavelength of 550 nm and a reference wave length of 630 nm. Each test was set up in duplicate and repeated three times. The IC_50 _value was determined by linear interpolation according to the method described in [27].

## Competing interests

The author(s) declare that they have no competing interests.

## Authors' contributions

K.M. carried out the experimental work as part of her VMD. D.S. conceived the study, supervised the execution, and prepared the final draft of the manuscript. Both authors read and approved the final manuscript.
